# "The Hospital" Medical Book Supplement—No. XXIX

**Published:** 1910-05-21

**Authors:** 


### "THE HOSPITAL" MEDICAL BOOK SUPPLEMENT—No. XXIX.

CONTENTS.

| | PAGE |
|---|---|
| **Medicine—** | |
| A Textbook of Physico-Therapy ... | 233 |
| Psychotherapeutics ... | 233 |
| Serums, Vaccines, and Toxins: in Treatment and Diagnosis... | 234 |
| Hypnotism and Treatment by Suggestion ... | 234 |
| Practical Pathology: a Manual for Students and Practitioners | 234 |
| La Thérapeutique des Affections Utéro-ovariennes par la Cure Chlorurée Sodique Bromo-Iodurée de Salies-de-Béarn ... | 234 |
| **Surgery—** | |
| Operations in General Practice ... | 235 |
| **Gynæcology—** | |
| Practical Obstetrics ... | 235 |
| **Diseases of Children—** | |
| The Diseases of Infants and Children ... | 235 |
| **Miscellaneous—** | |
| The Prayer Quest ... | 236 |
| A Code of Rules for the Prevention of Infectious and Contagious Diseases in Schools ... | 236 |
| Mind and Health ... | 236 |

### MEDICINE.

### A TEXTBOOK OF PHYSICO-THERAPY.*

It is but comparatively recently that any serious attempt has been made by medical observers to study the art of physico-therapy, or the treatment of disease by physical agents such as light, heat, water, electricity, massage, and graduated movements, active and passive. The therapeutic value of water, heat, and massage had been recognised for centuries before the Christian era, but it is only in the last few decades that any really scientific knowledge of their potentialities and limitations has been added to that possessed by the ancient empiricists. When it was realised that electricity could be applied to the treatment of disease, a great impetus was given to the study, from the medical standpoint, of that agent, the importance of which has been greatly increased by recent improvements in apparatus, and the discovery of new methods of application. The interest evoked in that agent had the effect also of reviving the study of other forms of physico-therapy, so that in time some kind of order was evolved out of the former empirical chaos, until at the present time the physician has at his disposal a number of well-tried methods of treatment other than by drugs. There can be little doubt that the importance of physico-therapy is as yet hardly realised by the profession in this country. The average medical student leaves his hospital after qualification with very little, if any, knowledge of the art. The hospitals themselves are only just beginning to be alive to the necessity of forming departments under the care of a skilled physician, where such treatment can be carried out. In private the medical profession has been forestalled by public trading companies, whose medical knowledge is as limited as their liability. For the most part these sumptuous establishments are presided over by electricians, and even such of them as claim to have the services of a medical man at the disposal of their clients are not above suspicion, in that it is usually difficult to find out both his name and qualifications. A book recently written by Dr. J. A. Rivière, of Paris, shows that in France some, at any rate, of the faculty are alive to the importance of physico-therapy. A pioneer in his own country, the inventor of numerous appliances and methods of treatment, and an ardent believer in the value of this art, the author, in his "Esquisses Cliniques," has collected together the fruits of an experience extending over twenty-five years. His first eleven chapters are devoted to the apparatus necessary for the application of the agents used, the rest of the book dealing with the various chronic disorders in which treatment by their means is of benefit. In the small amount of space at his disposal the author is, of course, unable to enter fully into the description of the complicated apparatus necessary for the art, but a series of photographs and diagrams to some extent supplies this deficiency. The book is readable, and should prove of interest to those who are concerned in this branch of the healing art. Its interest to the general practitioner lies not so much in the methods of application of this form of therapeutics, as in the directions given as to what classes of patients would be benefited thereby, for it is obvious that the erection of plant sufficient to carry out the various forms of physico-therapy would be commercially impossible except for the specialist. Moreover, the very exact knowledge, both of electricity and medicine necessary for proper treatment by physical agents is such that only the specialist could be expected to be possessed of it. If the book has the effect of drawing attention to, and increasing the interest in, a hitherto somewhat neglected branch of therapeutics, the author's purpose will have been served.

PSYCHOTHERAPEUTICS. A Symposium by Drs. PRINCE, GERRISH, PUTNAM, TAYLOR, SIDIS, WATERMAN, DONLEY, JONES, and WILLIAMS. (T. Fisher Unwin, Adelphi Terrace. 4s. 6d. net.)

REALLY it is time that medical men who venture to publish books should study the etymology of the language they use and know the meaning of the words they employ. The word "symposium" is always being ill-used by members of the profession, but that is no reason why we should not once more offer a protest against the manner in which these American gentlemen employ it and other words. "Psychotherapeutics" is a reissue of a series of papers that have appeared in the *Journal of Abnormal Psychology*, these

* "Esquisses Cliniques de Phisicotherapie." Par le Dr. J. A. Rivière, de Paris. Précédé d'une lettre du Proff. Lanceraux, et accompagné de 64 gravures. 8°, pp. 400. Prix 7 fr. 50c. (Paris: A. Maloine, éditeur 1910.).

papers being, again, extensions of addresses delivered before the American Therapeutic Society. The writers are well-known American specialists whose opinions are entitled to respect and consideration. Unfortunately they give us no opinions which as original dicta are in any way novel. We have read this little volume through without lighting upon any point that has been cleared up or discussed in a better or more able manner than has been done by English or Continental writers. Perhaps the most original and thoughtful address is that of Dr. Williams, of Washington, on "Psychoprophylaxis in Childhood," by which the author means "the preservation of health by psychic means." This brief chapter will well repay reading, for the author—unlike some of his predecessors—thinks clearly, and therefore expresses himself with a refreshing clarity of diction which is in striking contrast to the turgid and flatulent tone adopted by some of the other writers. Here and there we come across a sentence that grates, and at the end the reader has a lurking suspicion that Dr. Williams is not quite sure of the difference between monism and determinism! But his article is a useful and elucidative contribution to a subject that threatens to become hackneyed if more of these "symposia" are allowed to encumber an already alarmingly large literature on psychotherapy.

Serums, Vaccines, and Toxins: In Treatment and Diagnosis. By W. C. Bosanquet, M.A., M.D.Oxon., F.R.C.P., and John W. H. Eyre, M.D., M.S. Dunedin, F.R.S.Edin. (London: Cassell and Co. 7s. 6d.)

Perhaps in no branch of medicine has so important an advance been made as the treatment of various diseases by vaccines, serums, and toxins. Hence it is all-important that a good text-book on this subject should be obtainable, and we have no hesitation in saying that this revised edition of an already well-known work is one of the best that we know. It is five years since the first edition was published, but so many new theories have been advanced, so many new methods adopted in treating disease that a new edition was highly necessary in order that the book might be thoroughly up to date. Therefore much new matter has been added, and especially is this the case in describing the various treatments by vaccines that are so much in vogue just at present. The use of tuberculin as a remedy for tubercular diseases of all kinds has carefully been considered, and the revision of doses thoroughly gone into, and by this newer method it is hoped that the old dangers will be obviated. We have nothing but praise for this thoroughly up to date and useful treatise on one of the most interesting and fascinating subjects of the day in the medical world.

Hypnotism and Treatment by Suggestion. By J. Milne Bramwell, M.B., C.M. (London: Cassell and Co., Ltd. 5s. net).

To those who are in any way interested in the subject of hypnotism this book will appeal strongly. And even to those who do not believe in hypnotic power much food for thought is provided, for there is a great deal in the book that cannot be disregarded by the sceptics. The author has evidently put to the test all that he preaches, and some of the cases he cites as having received treatment by hypnotism and suggestion, and the results so obtained, are remarkable. We are especially interested in the chapter describing the surgical cases that have been dealt with by hypnosis. If to everyone power were given to render hypnotic those of their patients on whom some surgical operation was to be performed, then the use of the anæsthetics employed nowadays would, we think, gradually be replaced by this new art of anæsthetising. And if the danger of chloroform or any anæsthetic now used could be done away with, together with the too often unpleasant after-effects of nausea, what an advance on medical science would have been made! But there is distinctly another side to this most interesting question, and that is that hypnotism put to its various uses by an unscrupulous person could become a danger too colossal to imagine. The author deals with various forms of quackery now rampant, and gives as an instance the "Christian scientist," and he deals with it in a very clever and competent way, and as we read we understand that he is desirous of dissociating the science—for it is undoubtedly such—of hypnotism from any of these quackeries, and he would lay especial stress upon the dangers they evolve.

Practical Pathology: A Manual for Students and Practitioners. By G. Sims Woodhead, M.D., LL.D. Fourth Edition. (Henry Frowde, and Hodder and Stoughton, Warwick Square, E.C. 31s. 6d. net.)

As a student's manual this book is well known, though it has been out of print for many years. It is largely a class text-book, and though it deals with general pathology it concerns itself much more with laboratory and post-mortem-room work. The first chapter is devoted to the post-mortem examination, and the author advocates the use of his special blunt-pointed section knives as less liable to make punctured wounds. Those who are in the habit of making autopsies will find the ordinary knives fully as useful and as harmless, and the inexpert practitioner will not be safeguarded by the use of blunted instruments. The chapter is very well written and very full, and is specially to be recommended to students and practitioners as giving an excellent account of the way in which a post-mortem should be conducted. The rest of the book deals with special pathology. There are excellent illustrations, which really give a fair idea of what one expects to see under the microscope when examining a typical section. They are in colour, and thereby an additional advantage, to the student at least, is secured. Ordinary wood blocks showing blurred and indistinct pictures of what purport to be micro-photographs are not of the slightest utility in such a manual, and it is gratifying to observe that Professor Woodhead has discarded them in favour of these fine coloured delineations. Staining methods and essentials are briefly but comprehensively dealt with. Of the special sections the most interesting are those dealing with the nervous system and with bone and joint lesions, but those dealing with the pathology of lung and heart lesions are equally full and excellently written. The book is perhaps of far greater value to the student who is actually doing a course of pathological work than to the practitioner. The latter will, however, find that it is worth his while to possess a good modern work on practical pathology, and as such this volume, which is, like all the books of this firm, well printed and bound, may be confidently recommended to him.

La Thérapeutique des Affections Utéro-ovariennes par la Cure Chlorurée Sodique Bromo-Iodurée de Salies-de-Béarn. Par le Dr. David. (Paris: Octave Doin et fils. Editeurs 1910. Pamphlet of eight pages.)

The author believes that many cases of uterine and ovarian disease show no improvement after even the most energetic surgical and medical treatment, because too much attention is paid to the local disorder, while the general condition of the patient is neglected. Many chronic cases are cured at Salies-de-Béarn because treatment is directed towards the improvement of the general tone of the patients' organism by baths and the taking of the waters which contain, in addition to other drugs, bromides to the extent of 3½ grains to the pint, and are consequently sedative and useful in those cases where there is nervous instability. A series of cases are described in which this treatment led to most satisfactory results.

### SURGERY.

Operations in General Practice. By Edred M. Corner, M.C., F.R.C.S., and H. Pinches, M.B., B.C. Third Edition. "Oxford University Manuals." (Hodder and Stoughton, Warwick Square, E.C. 15s. net.)

The fact that this work has been so well received that a third edition is necessary within so short a time is a proof of the excellence of the book and its well-deserved popularity. As a manual eminently suited to the requirements of the general practitioner we have already reviewed it at some length, and can only repeat here that it is a volume that should be on the shelves in every surgery. The third edition has been revised and enlarged by the addition of a further chapter dealing with recent advances. We are glad that the authors do not any longer recommend elaborate dressings for skin grafts; their practice in leaving the grafted area exposed to the air and protected simply by a cradle from contamination with the bed-clothes is in accordance with the rule followed by many surgeons in this country, and is certainly superior to the old methods. A short description of Demars and Marx' operation for the relief of obesity is given; the authors do not say if they have had clinical experience of the operation, which, so far as we recollect at the moment, has not been performed in England. Schultz' recently published record of cases is certainly very encouraging, but it is doubtful if the operation can legitimately be classed under those of general practice. The authors, from their experience at Great Ormond Street, condemn the bismuth treatment of suppurating sinuses; certainly in practice far more troublesome sinuses are cured by the hyperæmic method than by such plugging. In the next edition an account of the use of iodine solution for sterilising the skin previous to incisions may usefully be added; in private practice this method is in our opinion far superior to any other. Two other additions we would suggest are descriptions of the solid $CO_2$ treatment of nævi and the use of radium applicators. We are by no means sure that an orthopædist of experience will like the authors' model boot on page 249, and indeed the orthopædic notes in the book stand in need of some revision. Thus the method of tenotomy advised is the old—and bad—way of doing the operation. It is natural, however, that the authors have confined themselves to one operation in each case. Such a book as this must necessarily be dogmatic, and its brevity forbids any discussion of the usefulness of alternative methods.

### GYNÆCOLOGY.

Practical Obstetrics. By E. Hastings Tweedy, F.R.C.P.I., and G. T. Wrench, M.D. Second Edition. "Oxford University Manuals." (Hodder and Stoughton, Warwick Square, E.C. 12s. 6d.)

Tweedy and Wrench has already become a popular work with students and practitioners alike, and this, the second edition of so useful a work, is to be welcomed, as it is in effect a full exposition of the Rotunda methods. There are many important revisions—notably in the chapter on "Toxæmia of Pregnancy"—and the authors have slightly modified their opinions, as expressed in the first edition, with regard to the treatment of ruptured uterus and contracted pelvis. Speaking for himself, Dr. Tweedy remarks that the book is a record of an experience of at least 30,000 cases for which he has been personally responsible. As such the practical value of the work is immense. Indeed, we know of no volume on obstetrics of the same size and price that has such a high practical value. Pubiotomy is highly spoken of and more fully described than is usually the case in text-books, Bumm's subcutaneous method being also briefly referred to. We cannot agree with the authors that this is the operation of choice in cases where half an inch increase in the brim conjugate makes all the difference in treatment—certainly not because it sometimes permanently increases the size of the pelvic brim. Where such permanent increase results it is due to serious weakening of the pelvic ligaments, as the excellent and systematic series of skiagraphs taken at the Charité Clinic of the cases operated upon by Bumm, Martin, and others has demonstrated. We have no desire to criticise the various methods of treatment described in this book. Dr. Tweedy's practical work is a sufficient guarantee that they are methods which are sound and useful, however contrary they may in some cases be to methods some of us favour. The main point is that the second edition is a thoroughly trustworthy practical guide, and as such it is eminently deserving the attention of student and practitioner.

### DISEASES OF CHILDREN.

The Diseases of Infants and Children. By Edmund Cautley, M.D., F.R.C.P. (London: Shaw and Sons. 1910. Pp. 1052. Price £1 1s.)

Among the largest and most ambitious treatises upon children's diseases those of American authors have hitherto been the most widely and deservedly popular. But there is no American monograph upon this subject at once so sound, so original, and so complete as this admirable production; and it will be surprising if it does not remain, as a work of reference and post-graduate text-book, the most consulted book on pediatrics for a very considerable time. The teaching is throughout practical; and diagnosis, prognosis, and treatment are always discussed at greater length than pathology or academic points of ætiology, as is fit and proper in a work designed for practising medical men. Dr. Cautley, indeed, expressly addresses himself in his preface to those in practice; and he has taken the quite unique course of excluding charts, illustrations, diagrams, and photographs of all kinds. He stigmatises all pictures as kindergarten methods of teaching medicine—useful, perhaps, to the student, but disadvantageous to the post-graduate. Whether he is right or not about this—very likely he is—it is undeniable that the extra space thus gained has been put to the very best advantage; the book is very heavy (avoirdupois) as it is, and to have added numerous illustrations must have involved either increase of weight or curtailment of the letterpress.

Dr. Cautley has a turn for paradox, to which he allows rein now and then, but with discretion. A happy instance of the ironical touch with which he can show up ignorance is to be found in the opening words of chapter xxx: "The appendix is either a vestigial remnant or a highly differentiated and specialised lymphoid structure." Another shaft of the same nature is launched in the statement that "the death rate of infants is lowest amongst the British Peerage and the fisher people of the Faröe islands." It is only here and there that he allows his fancy such play; but all through it is evident that he is an original thinker, and that mere compilation or the repetition of other people's ideas is far from satisfying his ideals of what a text-book should be. The arrangement of the chapters is good; the exposition both of general principles and of particular instances is clear and logical; and the general style, though not beyond the reach of a carping critic, is at least vigorous and interesting.

Where almost everything is excellent, it becomes difficult to select the best sections. On infant feeding, as might be expected, the author is both dogmatic, exhaustive, and explicit. He is well known already as an opponent of the "milk prescription"; some of the grounds of his objections as here set forth seem rather inadequate. The method which he describes of preparing a substitute food is certainly simple, and doubtless gives very good results. It is noteworthy that the same space (twenty pages) is allotted to the subject of breast-feeding as to that of substitute feeding—a just proportion. Diet after weaning also comes in for detailed consideration; this matter is too often slurred over, and both on this and on the lengthy discussion of breast-feeding Dr. Cautley is to be congratulated. Another point dealt with is the detection of chemicals added to milk; this also is most valuable. To the methods of preparing so-called humanised milk with whey much less attention is devoted; this term is used, moreover, to denote a partially peptonised milk which is recommended for the first three or four months of life in delicate children. The sections upon acidosis, cyclical vomiting, delayed chloroform poisoning, and similar mysterious disorders of metabolism are very careful; but the prophylactic administration of glucose before an anæsthetic, so highly spoken of by Wallace and Gillespie, is not mentioned, nor is sodium bicarbonate in the treatment of the established condition. As a minor point of interest, it may be mentioned that the usual etymology of rickets (Anglo-Saxon *wrikken*, to twist) is ignored, and preference given to the Norman-French *riquets*, deformities.

Dr. Cautley deprecates the usual custom of wiping the mouth of an infant, after feeding, with a rag dipped in water or boric-acid solution; this is, he says, unnecessary, and a common source of injury to the mucous membrane; undue cleaning is also said to favour thrush. He attaches less importance to phimosis as a cause of hernia than most authorities do; and the wool-truss receives emphatic condemnation. Operation is recommended for most cases in male infants, preferably after the third month and before weaning; but the author admits that many surgeons do not operate before four years of age owing to the frequency of spontaneous cure at one or two years old. A sentence out of the next chapter but one (on appendicitis) would, perhaps, apply here also: "If all patients are operated on, many cases which would recover by resolution are submitted to an unnecessary and expensive ordeal." Probably most surgeons would prefer conservatism in regard to the herniæ of infants rather than to their appendix troubles. The chapters on pulmonary diseases are very good. It is pleasant to find that Dr. Cautley disapproves of the practice of giving powerful (and therefore depressing) emetics to babies with the view of clearing their air passages of accumulated secretion. In general, he is not in favour of resection of rib for empyema in children under two, but prefers simple incision as adequate and less dangerous.

Erythema nodosum is described as an acute specific febrile disorder with an incubation period, prodromata, eruption, and stage of convalescence. Its conjectured association with rheumatism is denied, though the arthritis which is sometimes seen during the disease is admitted to resemble that of rheumatism in early life. This disease is classified among disorders of the blood and lymph, in the same chapter with hæmophilia. In this latter condition it is laid down that the female members of hæmophilic families should not bear children, and that the teeth of bleeders are not to be extracted; dissent from both these propositions is not improbable. For nocturnal incontinence it has of late years been fashionable to scrape the naso-pharynx; Dr. Cautley thinks the importance of adenoids in the causation of enuresis has been greatly exaggerated, and he is also not very enthusiastic about the thyroid extract cure which has lately been so much eulogised. The chapters on infective diseases are among the best in the volume. Dealing with the contagiousness of inherited syphilis, the author is in agreement with Dr. Still, Henoch, Gunzburg, and many others, who regard it as very slight; but Fournier and Sir J. Hutchinson are of the contrary opinion. Transmission to the third generation he regards as still not proven. The book concludes with adequate, but not of course exhaustive, accounts of the diseases of the eye, ear, skin, etc. It is a book which reflects great credit not only upon the author, but upon British medicine as a whole. Rarely is it possible to praise a bulky text-book so unreservedly; there can be no hesitation in recognising this as the premier book on children's diseases published in this country. It is only necessary to add that the publishers' share of the work has been done very well indeed.

### MISCELLANEOUS.

The Prayer Quest. By W. Winslow Hall, M.D. (London: Headley Brothers. 2s. 6d. net.)

We have had many strange books to review in our time, but perhaps none so strange as the present one. The Hospital does not profess to be a theological organ, and we cannot go into the points raised by the author, who appears to be a medical man and who expounds his views both in prose and in doggerel. The most merciful criticism that we can give his work is to remain silent about it.

A Code of Rules for the Prevention of Infectious and Contagious Diseases in Schools. Issued by the Medical Officers of Schools Association. (London: J. and A. Churchill. 1s. net.)

This present issue of the "Code" marks the sixth edition, and every chapter has been thoroughly revised and brought up to date, and much new matter has been introduced. It is undoubtedly one of the most valuable aids for the prevention of the spread of all kinds of infectious diseases that has been written, and no medical officer, in whatever capacity he may be working, should be without this helpful little guide. Especially valuable is the Appendix C, which treats of disinfection in all its various forms. It is well got-up, the type is clear, and the price moderate.

Mind and Health. By Edwin Ash, M.D. (London: H. J. Glaisher. 2s. 6d. net.)

Within quite recent years the treatment of various nervous diseases, and especially that of neurasthenia, has been increasingly by suggestion. The author has endeavoured in this little book to expound more fully the "mental therapeutics," as he is pleased to call them, and to indicate how best the mind can govern the body to the extent of aiding the recovery of a patient suffering from some functional nervous disorder. The chapter on neurasthenia is the best one. It is carefully written, and in many respects is well thought out, though we do not find ourselves in accord with all that is said. The writer has evidently spent much time and trouble on this book, and those interested in any way in this subject may find it worth their while to read it.

